# Lymphangiome kystique de l'arrière-cavité des épiploons

**DOI:** 10.11604/pamj.2014.17.48.2422

**Published:** 2014-01-23

**Authors:** Rachid El Barni, Mohamed Lahkim, Jawad Fassi Fihri, Abdelhadi Mejdane, Rachid Bouchama, Abdessamad Achour

**Affiliations:** 1 Service de chirurgie générale, Hôpital militaire Avicenne, Marrakech

**Keywords:** Lymphangiome kystique, arrière-cavité des épiploons, chirurgie, cystic hygroma, lesser sac, surgery

## Abstract

Le lymphangiome kystique de l'arrière-cavité des épiploons est une localisation exceptionnelle dont le diagnostic est suspecté par la radiologie et confirmé par l'examen anatomo-pathologique. L'exérèse chirurgicale constitue le traitement de choix.

## Introduction

Le lymphangiome kystique est une tumeur bénigne et rare des vaisseaux lymphatiques. Elle est exceptionnelle chez l'adulte [1] et siège plus particulièrement au niveau du cou et du creux axillaire (95%) [2]. Les formes intra-abdominales sont rares de l'ordre de deux à 10% [1] et se situent préférentiellement dans le mésentère et le rétropéritoine [3]. Le siège sus-mésocolique est exceptionnel [4]. Les auteurs rapportent une nouvelle observation de lymphangiome kystique de l'arrière-cavité des épiploons (ACE).

## Patient et observation

**Figure 1:**
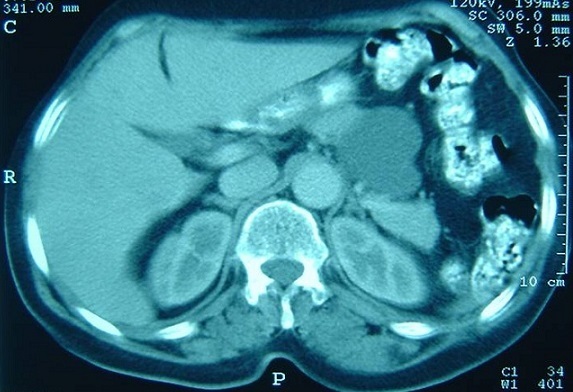
Tomodensitométrie abdominale (coupe transversale): une masse kystique liquidienne et uniloculaire, située au contact de la région corporéo-caudale du pancréas

**Figure 2:**
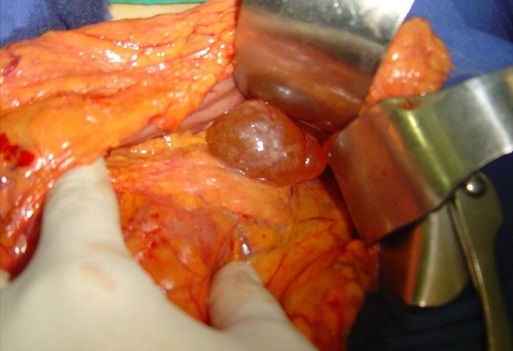
Vue peropératoire: une masse kystique uniloculaire à paroi translucide et à contenu purement liquidien, prolabée dans l'ACE

**Figure 3:**
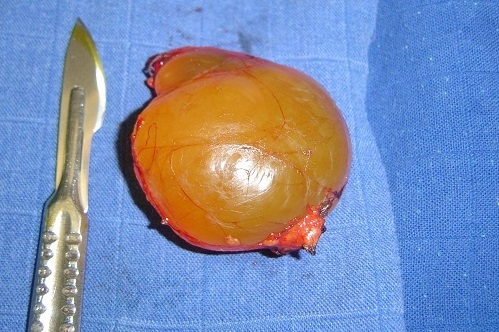
Pièce de résection du lymphangiome kystique

**Figure 4:**
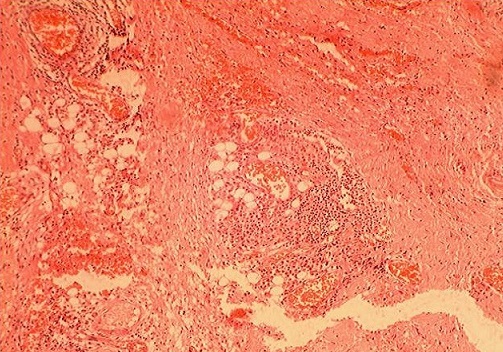
Coupe histologique de la pièce opératoire montrant des lumières pseudokystiques de différente taille, bordées de cellules endothéliformes aplaties

Mme F. Z, âgée de 63 ans, sans antécédent pathologique particulier, présentait des douleurs épigastriques évoluant depuis trois mois dans un contexte de conservation de l’état général. L'examen clinique trouvait une légère sensibilité de la région épigastrique sans masse palpable. La fibroscopie Œso-gastroduodénale était normale. L’échographie et la tomodensitométrie abdominales objectivaient une masse kystique, uniloculaire, mesurant 49x45 mm, à paroi fine et régulière, et à contenu totalement liquidien, sans cloison ni végétations endokystiques. Cette masse, ne présentant pas de rehaussement pathologique après injection du produit de contraste, se prolabait dans l'ACE et exerçant un effet de masse sur la région corporéo-caudale du pancréas (Figure 1). La biologie ainsi que les marqueurs tumoraux étaient normaux. La sérologie hydatique était négative. Au terme de ce bilan, le diagnostic d'une tumeur kystique bénigne de l'ACE était retenu. Une laparotomie exploratrice était décidée, et le décollement colo-épiploïque avait permis de trouver une masse kystique uniloculaire à paroi translucide et à contenu purement liquidien, mesurant 5 cm de grand axe, située dans l'ACE au contact du corps du pancréas (Figure 2). Une exérèse complète sans sacrifice pancréatique était réalisée (Figure 3). L'examen anatomopathologique de la pièce opératoire, mesurant 5x5x3 cm, trouvait à la coupe un aspect kystique avec issu d'un liquide clair et filant. L'analyse microscopique de la coupe réalisée montrait des lumières pseudokystiques, tortueuses et de différente taille, bordées par des travées conjonctives abritant ici et là des traînées lymphoïdes et alternant avec quelques vaisseaux capillaires congestifs. Les cellules bordantes sont aplaties, endothéliformes. Le contenu luminal est légèrement éosinophile, abritant essentiellement des lymphocytes (Figure 4). Les suites opératoires étaient simples et l’évolution était favorable sans récidive avec un recul de 3 ans.

## Discussion

Le lymphangiome kystique abdominal est une tumeur bénigne et rare du système lymphatique. La symptomatologie clinique est polymorphe et non spécifique, elle peut résulter soit du volume tumoral (douleur abdominale, compression), soit c'est une symptomatologie bruyante lors d'une complication à type de rupture, infection, hémorragie intra-kystique, occlusion, torsion, compression ou infiltration des structures vitales [5]. La transformation maligne est exceptionnelle [6]. Le diagnostic est évoqué par la radiologie et confirmé par l’étude histologique. Le traitement de choix est chirurgical et consiste en une exérèse complète de la lésion évitant ainsi les récidives. Le pronostic reste bon.

## Conclusion

Le lymphangiome kystique est une tumeur bénigne d'origine lymphatique dont la clinique est polymorphe. Le diagnostic est suggéré par l'imagerie et confirmé par l'histologie. La chirurgie d'exérèse est le seul traitement permettant d’éviter les récidives locales.
